# Comprehensive Enzymatic Analysis of the Cellulolytic System in Digestive Fluid of the Sea Hare *Aplysia kurodai*. Efficient Glucose Release from Sea Lettuce by Synergistic Action of 45 kDa Endoglucanase and 210 kDa ß-Glucosidase

**DOI:** 10.1371/journal.pone.0065418

**Published:** 2013-06-06

**Authors:** Akihiko Tsuji, Keiko Tominaga, Nami Nishiyama, Keizo Yuasa

**Affiliations:** Department of Biological Science and Technology, The University of Tokushima Graduate School, Tokushima, Japan; Universidad de Granada, Spain

## Abstract

Although many endo-ß-1,4-glucanases have been isolated in invertebrates, their cellulolytic systems are not fully understood. In particular, gastropod feeding on seaweed is considered an excellent model system for production of bioethanol and renewable bioenergy from third-generation feedstocks (microalgae and seaweeds). In this study, enzymes involved in the conversion of cellulose and other polysaccharides to glucose in digestive fluids of the sea hare (*Aplysia kurodai*) were screened and characterized to determine how the sea hare obtains glucose from sea lettuce (*Ulva pertusa*). Four endo-ß-1,4-glucanases (21****K, 45****K, 65****K, and 95****K cellulase) and 2 ß-glucosidases (110****K and 210****K) were purified to a homogeneous state, and the synergistic action of these enzymes during cellulose digestion was analyzed. All cellulases exhibited cellulase and lichenase activities and showed distinct cleavage specificities against cellooligosaccharides and filter paper. Filter paper was digested to cellobiose, cellotriose, and cellotetraose by 21****K cellulase, whereas 45****K and 65****K enzymes hydrolyzed the filter paper to cellobiose and glucose. 210****K ß-glucosidase showed unique substrate specificity against synthetic and natural substrates, and 4-methylumbelliferyl (4MU)-ß-glucoside, 4MU–ß-galactoside, cello-oligosaccharides, laminarin, and lichenan were suitable substrates. Furthermore, 210****K ß-glucosidase possesses lactase activity. Although ß-glucosidase and cellulase are necessary for efficient hydrolysis of carboxymethylcellulose to glucose, laminarin is hydrolyzed to glucose only by 210****K ß-glucosidase. Kinetic analysis of the inhibition of 210****K ß-glucosidase by D-glucono-1,5-lactone suggested the presence of 2 active sites similar to those of mammalian lactase-phlorizin hydrolase. Saccharification of sea lettuce was considerably stimulated by the synergistic action of 45****K cellulase and 210****K ß-glucosidase. Our results indicate that 45****K cellulase and 210****K ß-glucosidase are the core components of the sea hare digestive system for efficient production of glucose from sea lettuce. These findings contribute important new insights into the development of biofuel processing biotechnologies from seaweed.

## Introduction

Lignocellulosic waste materials obtained from energy crops, wood, and agricultural residues represent the most abundant global source of renewable biomass [1]–[3]. However, the task of hydrolyzing lignocellulose to fermentable monosaccharides is technically problematic. Lignocellulose is composed of 3 major constituents: cellulose, hemicellulose, and lignin. The crystallinity of cellulose, hydrophobicity of lignin, and encapsulation of cellulose by the lignin–hemicellulose matrix are 3 major factors that contribute to the recalcitrance of lignocellulose. Unlike sucrose or starch, lignocellulosic biomasses need pretreatment such as steam explosion to make cellulose accessible for efficient enzymatic depolymerization [4], [5]. While the quantity of enzymes needed to produce ethanol from starch-based feedstock is very low, the complexity of cellulosic-based feedstocks can require as much as 100 times the amount of enzymes needed for starch conversion. The hydrolysis of cellulose to glucose catalyzed by cellulases is a key step in the efficient production of biofuel from lignocellulosics. *Trichoderma reesei* is currently the principal source of commercial cellulase [2], [6], [7]. The synergistic action of different types of cellulolytic enzymes (ß-1,4-endoglucanase, cellobiohydrolase, and ß-glucosidase) is required for effective cellulose hydrolysis [8]. However, large amounts of cellulases are still required for efficient decomposition of biomass, and this increases the cost of biofuel production.

On the other hand, there is increasing interest in algae as one of the alternative renewable sources of biomass for production of bioethanol, which is considered “third-generation biofuel” [9], [10]. In comparison to other feedstocks, algae can be used as a high-yield source of biofuels without compromising food supplies, rainforests, or arable land. Furthermore, bioethanol from algae holds significant potential because of their low percentage of lignin and hemicellulose in comparison with other lignocellulosic biomasses. Pretreatment for the solubilization of seaweed polysaccharides is less severe than that required for lignocellulosic materials. Seaweeds (macroalgae) are fast-growing marine plants, and the growth rates and yields per surface area that can be obtained from seaweed forests are significantly higher than those reported for terrestrial plants. Sea lettuce (*Ulva pertusa*) is a nuisance species of green algae and is found all over the world. Recent nutrient enrichment of coastal waters is leading to an increase in the growth rate of sea lettuce, and it is now developing into a serious environmental pollution problem. The effective use of sea lettuce waste as biomass is expected for a long time [11].

Sea lettuce is consumed by a number of sea animals. In particular, an East-Asian marine gastropod, the sea hare (*Aplysia kurodai*), shows a clear preference for sea lettuce, feeding well on the lettuce but rejecting *Gelidium* and *Pachydictyon* [12]. Investigation of sea hare digestion may therefore provide useful clues for establishment of an artificial process for saccharifying polysaccharides in sea lettuce. Although individual ß-1,4-endoglucanases have been purified from various marine animals, including crustaceans [13], [14], mollusks [15]–[18], and sea urchins [19], entire glucose production systems from polysaccharides in algae are yet to be clarified in higher animals, unlike in bacteria [20] and fungi [6]. Genome analysis and PCR cloning of glucanases using primers corresponding to conserved amino acid sequences show the existence of mRNA for a novel enzyme and elucidates the genetic evolution of glucanase [21]–[23]. However, information regarding cleavage specificity and the synergistic action of cellulolytic enzymes has not been obtained by these genetic approaches. Comprehensive functional analysis of glucanases in digestive fluid using purified enzymes is essential to clarify an efficient saccharification system [24], [25]. It is easier to prepare a sufficient amount of digestive fluid from the sea hare for purification of large quantities of glucanases than from other cellulase-producing animals. In this study, we purified 4 ß-1,4-endoglucanases and 2 ß-glucosidases from the digestive fluid of the sea hare at the mg level and analyzed their cleavage specificity, synergistic action on substrates, and glucose-producing activity from seaweed. Our findings provide the first example of an enzymatic process of glucose liberation from seaweed in digestive fluid of the sea hare.

## Results

### Purification of Cellulolytic Enzymes from the Digestive Fluid of the Sea Hare

**Figure 1:**
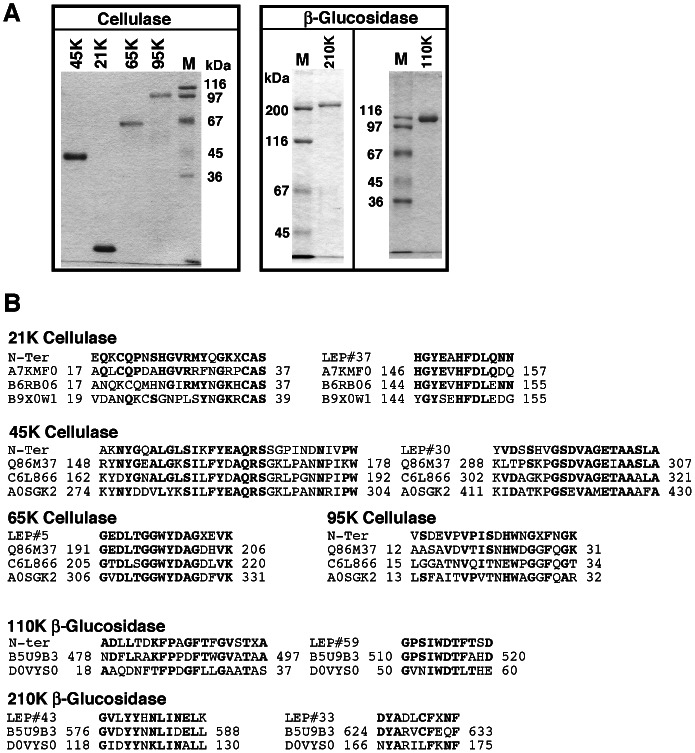
SDS-PAGE and amino acid sequence of purified enzymes. (**A**) SDS-PAGE of purified enzymes (2 µg protein). The marker proteins were as follows: myosin heavy chain (200 kDa), ß-galactosidase (116 kDa), phosphorylase b (97 kDa), BSA (67 kDa), ovalbumin (45 kDa), and glyceraldehyde-3-phosphate dehydrogenase (36 kDa). (**B**) Alignment of N-terminal and internal sequences of purified enzymes with other endo-ß-1,4-glucanases from freshwater snail (UniProt: A7KMF0, A0SGK2), brackish water clam (B9X0W1), abalone (B6RB06, Q86M37), and scallop (C6L866) and ß-glucosidases from brackish water clam (B5U9B3) and termite (D0VYS0). The molecular mass of A7KMF0, B6RB06, B9X0W1, Q86M37, C6L866, A0SGK2, B5U9B3, and D0VYS0 is 19 kDa, 21 kDa, 22.6 kDa, 66 kDa, 64 kDa, 66 kDa, 110 kDa, and 55 kDa, respectively. The internal sequences of fragments (LEP#37 from 21****K cellulase, LEP#30 from 45****K cellulase, LEP#5 from 65****K cellulase, LEP#59 from 110****K ß-glucosidase, LEP#33 and 43 from 210****K ß-glucosidase) generated by lysyl endopeptidase digestion of purified enzymes were determined as described in Materials and Methods. The amino acid residue numbers of other endo-ß-1,4-glucanases and ß-glucosidases are indicated on both sides of the corresponding sequences.

Four cellulases (21****K, 45****K, 65****K and 95****K) and 2 ß-glucosidases (110****K and 210****K) were purified from digestive fluid of the sea hare, as described in Materials and Methods. The procedures used for purification of the 4 cellulases and 2 ß-glucosidases are shown in Figure S1. The 21****K, 45****K, and 65****K cellulase found in the CM-Sepharose bound fraction were purified using phenyl-Sepharose (Figure S2, upper) and gel filtration (Figure S2 lower). 95****K cellulase and 210****K and 110****K ß-glucosidase found in the CM-Sepharose unbound fraction were purified using DEAE-Sepharose (Figure S3A), phenyl-Sepharose, and gel filtration (Figure S3B, 3C). The 210****K and 110****K ß-glucosidase were further purified using hydroxyapatite (Figure S4A) and Mono-Q (Figure S4B), respectively. As shown in Figure S4B, 210****K ß-glucosidase is active against 4MU-ß-glucoside and laminarin. Final preparations yielded a single protein band on SDS-PAGE [26] (Figure 1A), and the molecular mass of these enzymes was estimated as 21 kDa, 45 kDa, 65 kDa, 95 kDa, 110 kDa, and 210 kDa by SDS-PAGE. Specific activities of the purified 21****K, 45****K, 65****K, and 95****K cellulase toward CMC were 0.72, 17.1, 5.21, and 0.316 µmol/min/mg, respectively. Specific activities of 210****K and 110****K ß-glucosidase toward 4-MU-ß-glucoside were 69.9 and 15.5 µmol/min/mg, respectively. Considering the protein content and enzyme activity, 45****K cellulase may be the most abundant cellulase in the digestive fluid of the sea hare (Figure S1).

### Sequence Analysis

Alignment of N-terminal and internal amino acid sequences of the purified enzymes with endo-ß-1,4-glucanase from freshwater snail (UniProt ID: A7KMF0, A0SGK2) [17], [18], abalone (B6RB06, Q86M37) [15], Japanese scallop (C6L866), and brackish water clam (B9X0W1) [27] as well as ß-glucosidase from brackish water clam (B5U9B3) [23] and termite (D0VYS0) [28] are shown in Figure 1B. All sequences of 21****K, 45****K, 65****K, and 95****K cellulase and 110****K and 210****K ß-glucosidase were similar to the corresponding regions of other endo-ß-1,4-glucanases and ß-glucosidases, respectively. The internal sequence (LEP#37) of 21****K cellulase was highly homologous with a corresponding sequence of endo-ß-1,4-glucanase composed of about 200 amino acids from freshwater snail (A7KMF0) and abalone (B6RB06) belonging to the glycoside hydrolase family (GHF) 45 [29], [30]. The sequences of 45****K and 65****K cellulase were also highly homologous with a corresponding region of 65–66 kDa endo-ß-1,4-glucanases from abalone (Q86M37) [15], scallop (C6L866), and snail (A0SGK2) [18] belonging to GHF 9. The sequence FYEAQRS in the N-terminal sequence and internal sequence GSDVAGETAA (LEP#30) of 45****K cellulase as well as GEDLGGWYDAG (LEP#5) of 65****K cellulase are highly conserved sequences among GHF 9 family members. The N-terminal sequence of 95****K cellulase was homologous with a corresponding region of endo-ß-1,4-glucanase from abalone (Q86M37, amino acid identity: 50%), scallop (C6L866, 33%), and snail (A0SGK2, 40%). The internal sequence (LPAQNRIPYRGDS) of 95****K cellulase also showed similarity with a corresponding region of abalone (Q86M37, amino acid numbers 170–182, amino acid identity: 69%), scallop (C6L866, 184–196, 54%), and snail (A0SGK2, 296–308, 85%) endo-ß-1,4-glucanases.

The N-terminal and internal sequences of 110****K and 210****K ß-glucosidase were homologous with ß-glucosidase from brackis water clam [23] and termite [28] belonging to GHF 1. The internal sequences of 110****K (LEP#59, GPSIWDTFTSD) and 210****K (LEP#33, DYADLCFXF) were also homologous with corresponding regions represented by amino acid numbers 927–937 (**GPSIWD**N**FT**H, identical amino acids are in bold) and 1043–1053 (**D**S**YAD**F**CF**QT**F**) of human lactase-phlorizin hydrolase (LPH, often referred to simply as lactase) [31], respectively. A 110 kDa ß-glucosidase belonging to GHF 3 was recently purified from *Ustilago esculenta* and cloned [32]. However, the sequences of 110****K and 210****K ß-glucosidase did not show similarity with the amino acid sequence of this fungal ß-glucosidase. We found a similar but not identical internal sequence between 110****K and 210****K ß-glucosidase. The internal sequences LEP#59: **GPSIWD**T**F**TSD and LEP#60: **GV**NF**Y**NT**LI**DK of 110****K ß-glucosidase are similar to the internal sequence (identical amino acids are in bold) LEP#37: **GPSIWD**D**F**EH and LEP#43: **GV**LY**Y**HN**LI**NELK of 210****K ß-glucosidase, respectively. These results strongly suggest that 110****K ß-glucosidase is not a processed or degraded form of 210****K ß-glucosidase.

### Analysis of Oligosaccharides Bound to the Enzymes

To determine whether these purified enzymes are glycoproteins, the total hexose content of cellulases and ß-glucosidases was determined using the phenol-sulfuric acid methods [33]. Hexose was detected in all purified cellulases and ß-glucosidases. The total hexose content of 21****K, 45****K, 65****K, and 95****K cellulase as well as 110****K and 210****K ß-glucosidase was 167, 32.3, 252, 99.2, 66.3, and 66.9 µg glucose/mg protein, respectively. Furthermore, the structures of oligosaccharides bound to the enzymes were analyzed by lectin blot (Figure S5). Eight lectins comprising ConA (concanavalin A), PNA (peanut agglutinin), WGA (wheat germ agglutinin), LCA (lentil agglutinin), DBA (horse gram agglutinin), RCA-120 (caster bean agglutinin), PHA (phytohemagglutinin), and UEA (common gorse agglutinin) were used. WGA-reactive oligosaccharides were detected in 210****K and 110****K ß-glucosidase and in 95****K and 65****K cellulase. In addition, 210****K and 110****K ß-glucosidase reacted with ConA and LCA, while 95****K cellulase reacted with LCA but not ConA, and 65****K cellulase reacted with both PNA and LCA. Ovalbumin, one of the marker proteins, reacted with WGA and ConA. The cellulases and ß-glucosidases did not react with the DBA, PHA, RCA, or UEA lectins. Furthermore, 45****K and 21****K cellulase did not react with any lectins examined. These results indicate that 21****K, 45****K, 65****K, and 95****K cellulase as well as 210****K and 110****K ß-glucosidase are glycoproteins. Lectin blot analysis suggested that the oligosaccharide structures of these cellulases differed, whereas the oligosaccharide structures of the 2 ß-glucosidases were similar. These results indicate that none of these cellulases and ß-glucosidases are derived from gut-resident bacteria.

### Enzymatic Properties of Purified Cellulases and ß-glucosidases

All cellulases exhibited optimal hydrolytic activity of CMC at pH 5.0–5.5, while the optimal pH for 110****K and 210****K ß-glucosidase was pH 5.5–6.0. The digestive fluid of the sea hare has a pH of 5.5, which is very close to the optimal pH of all these enzymes. The optimal temperature for all enzymes, except 21****K cellulose, was around 40°C, and their activities decreased rapidly at temperatures above 50°C. In contrast, 21****K cellulase showed the highest activity at 50–55°C and showed 20% reduction in activity at 65°C. Both 110****K and 210****K ß-glucosidase were stable up to 55°C.

**Figure 2:**
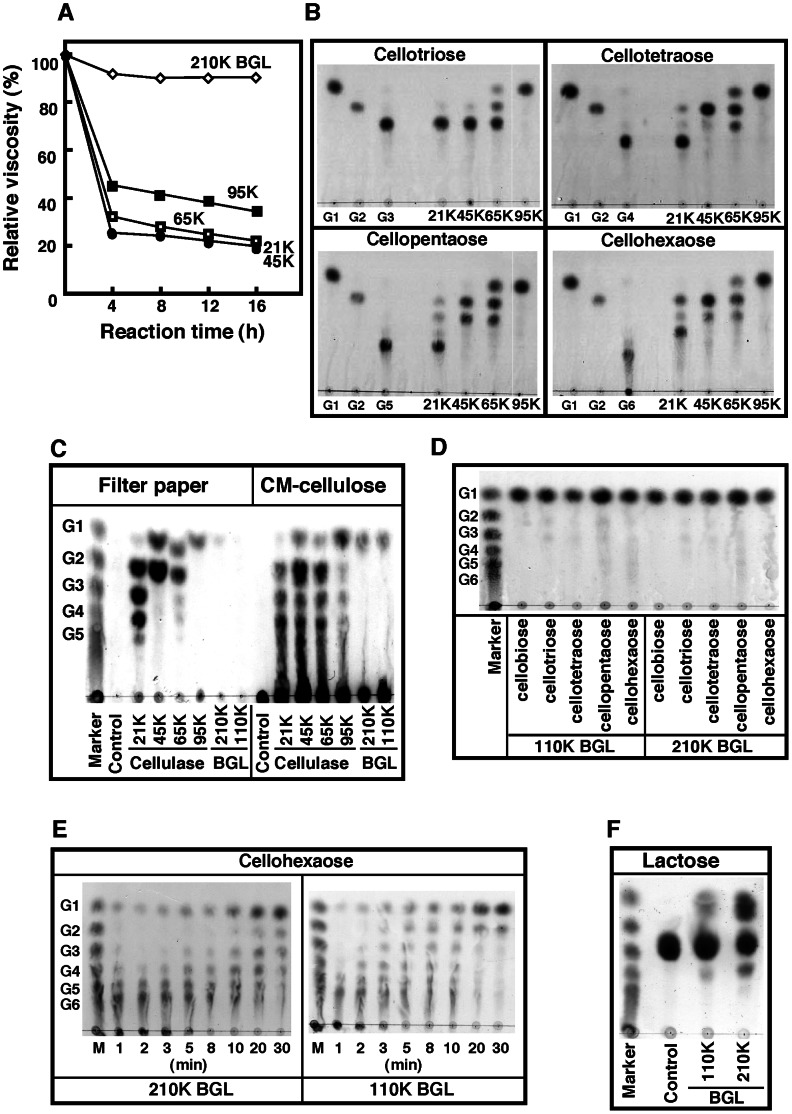
Mode of action of purified cellulases and ß-glucosidases from sea hare. (**A**) Effect of treatment with purified cellulases or 210****K ß-glucosidase on CMC viscosity. CMC (2 mL, 40 mg/mL in 50 mM acetate, pH 5.5) was incubated at 37°C for 4, 8, 10, 12, and 24 h in the absence or presence of the enzyme (0.2 µg) and the viscosity of the CMC solution was then measured as described in Materials and Methods. (**B**) Degradation of cello-oligosaccharides by purified cellulase. Cello-oligosaccharides (50 mL, 20 mg/mL in 20 mM acetate buffer, pH 5.5) were incubated with the enzyme (0.1 µg) at 37°C for 24 h and then subjected to TLC as described in Materials and Methods. G1, glucose; G2, cellobiose; G3, cellotriose; G4, cellotetraose; G5, cellopentaose; G6, cellohexaose. (**C**) Degradation of filter paper and CMC by purified cellulase and ß-glucosidases. Filter paper (60 mg) was incubated with 10 µg of the purified enzyme at 37°C for 15 h in 1 mL of 50 mM acetate buffer (pH 5.5). Furthermore, 1% CMC in 1 mL of 50 mM acetate buffer (pH 5.5) was incubated with the purified enzymes (2 µg) at 37°C for 1 h. The reaction mixture (2 mL) was subjected to TLC. (**D**) Digestion of cello-oligosaccharides with 210****K or 110****K ß-glucosidase. Cello-oligosaccharides (50 µL, 2 mg/mL in 10 mM acetate buffer, pH 5.5) were incubated with the enzyme (0.2 µg) at 37°C for 4 h and then subjected to TLC. (**E**) Time-course of hydrolysis of cellohexaose by the enzyme. Purified ß-glucosidase (BGL, 0.2 µg) was incubated with cellohexaose (50 µL, 20 mg/mL in 20 mM acetate, pH 5.5) for the time indicated. (**F**) Hydrolysis of lactose with 210****K or 110****K ß-glucosidase. Lactose (50 µL, 20 mg/mL in 20 mM acetate, pH 5.5) was incubated with the enzyme (0.2 µg) at 37°C for 4 h.

To determine the mode of action of cellulases in hydrolyzing CMC, the effect of enzymatic activity on the viscosity of CMC was first analyzed (Figure 2A). The viscosity of the CMC solution after enzyme treatment decreased rapidly in the first 4 h and then decreased slowly thereafter, suggesting that 21****K, 45****K, 65****K, and 95****K cellulase are endo-type glycosidases. In contrast, there was no change in the viscosity of the CMC solution treated with 110****K or 210****K ß-glucosidase, suggesting that these enzymes are exo-type glycosidases.

### Cleavage Specificity of 21****K, 45****K, 65****K, and 95****K Cellulase

The substrate specificities of cellulases were examined using various natural and synthetic substrates. Km and Vmax values toward CMC and lichenan for cellulases are shown in Table 1. Vmax values toward lichenan for all cellulases are higher than those toward CMC. Among the cellulases, 45****K cellulase showed the highest Vmax values for both CMC and lichenan. Yeast ß-glucan, laminarin, starch, and locust bean gum were not hydrolyzed by any of the cellulases.

**Table 1:** Enzyme activities toward CMC and lichenan of 21****K, 45****K, 65****K, and 95****K cellulase.

	Substrate					
Cellulase	CMC			Lichenan		
	Km %	Vmax µmol/min/mg	Vmax/Km	Km %	Vmax µmol/min/mg	Vmax/Km
21****K	2.65±0.21	1.89±0.02	0.71	2.11±0.11	4.64±0.09	2.19
45****K	8.13±1.40	90.6±5.60	11.1	3.21±0.32	128±17	39.8
65****K	1.20±0.07	12.6±0.47	10.5	4.27±0.57	91.6±1.23	21.5
95****K	1.37±0.23	0.649±0.08	0.47	3.06±0.2	5.00±0.42	1.63

The enzyme activity of all cellulases toward cello-oligosaccharides was also examined (Figure 2B). The cellulases displayed distinct cleavage specificities toward cello-oligosaccharides. None except 95****K cellulase was able to hydrolyze cellobiose. 21****K and 45****K cellulase had no activity toward cellotriose, whereas cellotriose was partially hydrolyzed to cellobiose and glucose by 65****K cellulase. 95****K cellulase hydrolyzed cellotriose to glucose. 21****K cellulase showed very weak activity toward cellotetraose and cellopentaose. These oligosaccharides were partially hydrolyzed by 21****K cellulase to cellotriose and cellobiose, whereas cellohexaose was completely hydrolyzed to cellotetraose and cellobiose. A trace amount of cellotriose was also detected. These results suggest that 21****K cellulase recognizes cellulose by the 6 units of glucose and possesses cellobiohydrolase activity in addition to endo-ß-1,4-glucanase activity. 45****K cellulase hydrolyzed cellotetraose to cellobiose, cellopentaose to cellobiose and cellotriose, as well as cellohexaose to cellobiose as a major product and cellotriose as a minor product. These results suggest that 45****K cellulase recognizes cellulose by the 4 units of glucose and possesses cellobiohydrolase activity in addition to endo-ß-1,4-glucanase activity. 65****K cellulase hydrolyzed cellotetraose, cellopentaose, and cellohexaose to cellotriose, cellobiose, and glucose. Cellotriose was partially hydrolyzed to cellobiose and glucose by 65****K cellulase. These results suggest that 65****K cellulase recognizes cellulose by the 3 (or 4) units of glucose and possesses cellobiohydrolase and ß-glucosidase activities in addition to endo-ß-1,4-glucanase. 95****K cellulase hydrolyzed cellotetraose, cellopentaose, and cellohexaose to glucose, suggesting that 95****K cellulase possesses ß-glucosidase activity in addition to endo-ß-1,4-glucanase.

The reaction products of filter paper and CMC after treatment with 21****K, 45****K, 65****K, and 95****K cellulase were also analyzed (Figure 2C). Cellotetraose, cellotriose, and cellobiose were released as major products by the digestion of filter paper with 21****K cellulase. Both 45****K and 65****K cellulase digested filter paper to cellobiose and glucose. A glucose monomer was detected in the reaction mixture of 95****K cellulase. 21****K cellulase digested CMC to cello-oligosaccharides (cellobiose, cellotriose, and cellotetraose as major products), and a glucose monomer was rarely detected. 45****K and 65****K cellulase digested CMC to cello-oligosaccharide (cellobiose as a major product) and had a glucose monomer. 95****K cellulase digested CMC to a glucose monomer as a major product and to cello-oligosaccharides as minor products.

### Cleavage Specificity of 110****K and 210****K ß-glucosidase

**Figure 3:**
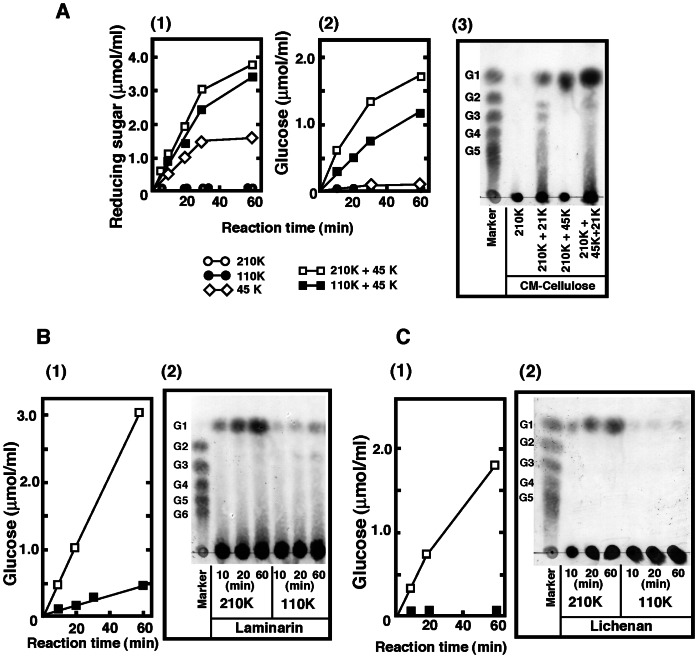
Mode of action of 210 K or 110 K ß-glucosidase on CMC, laminarin, and lichenan. (**A**) Digestion of CMC with 210 K or 110 K ß-glucosidase in the absence or presence of 21 K or 45 K cellulase. CMC (1 mL, 1% in 50 mM acetate, pH 5.5) was incubated with 2 µg of 210 K or 110 K ß-glucosidase and 5 mg of 21 K or 45 K cellulose, as indicated, at 37°C for 10, 20, 30, and 60 min. Reducing sugar (1) and glucose (2) in the reaction mixture were determined. Reaction products were analyzed by TLC (3). (**B**) Laminarin (1 mL, 1% in 50 mM acetate, pH 5.5) was incubated with 2 µg of 110 K or 210 K ß-glucosidase at 37°C for 10, 20, and 60 min. The glucose content in the reaction mixture was then determined. Reaction products were analyzed by TLC. (**C**) Lichenan (1 mL, 1% in 50 mM acetate, pH 5.5) was incubated with 2 µg of 110 K or 210 K ß-glucosidase at 37°C for 10, 20, and 60 min.

The cleavage specificity of 110****K and 210****K ß-glucosidase toward various synthetic and natural substrates was compared. Specific activity of both ß-glucosidases toward 4MU-ß-glucoside, 4MU-ß-galactoside, cellobiose, lichenan, and laminarin is shown in Table 2. 210****K ß-glucosidase exhibited higher specific activities against all substrates examined than 110****K ß-glucosidase. Among the synthetic substrates examined, both ß-glucosidases were most active against 4-MU-ß-glucoside. Compared with the maximum activity toward 4MU-ß-glucoside, 110****K and 210****K ß-glucosidase exhibited 34–35% activity toward 4-MU-ß-galactoside. Both ß-glucosidases showed weak activity toward 4MU-ß-xyloside (110****K: 0.9%, 210****K: 3.5%). Neither the 110****K nor the 210****K enzyme exhibited activity toward 4MU-α-glucoside, 4MU-α-galactoside, or 4MU-α-mannoside. Only a small amount of glucose was released from CMC by these ß-glucosidases (Figure 2C). All cello-oligosaccharides were completely hydrolyzed to glucose by both ß-glucosidases (Figure 2D). To investigate the mode of hydrolysis of cellohexaose, the time-course of degradation of cellohexaose by 110****K or 210****K ß-glucosidase was analyzed (Figure 2E). Cellohexaose was hydrolyzed into cellopentaose, cellotetraose, cellotriose, cellobiose, and glucose within 20 min (210 K) and 5 min (110 K). These results suggest that 210 K ß-glucosidase prefers longer oligo-cellulose than cellotriose. It is noteworthy that both enzymes exhibited weak lactase (Figure 2F) and gentiobiose cleavage activities (data not shown). Figure 3 shows the glucose-releasing activity of 110 K and 210 K ß-glucosidase toward CMC, laminarin, and lichenan. Lichenan is a ß-D-glucan that consists of repeating glucose units linked by ß-1,4 and ß-1,3 glycosidic bonds. Laminarin is a storage polysaccharide of seaweed consisting of a ß-1,3-linked glucose main chain and ß-1,6-linked glucose branches. Although collaboration between cellulases is necessary for production of glucose from CMC by ß-glucosidase (Figure 3A), laminarin and lichenan were efficiently digested to glucose only by 210 K ß-glucosidase (Figure 3B, 3C). Glucose release from laminarin and lichenan was confirmed by a quantitative glucose assay using glucose oxidase. Cardran, a polysaccharide consisting of ß-1,6-linked glucose, was also cleaved by both ß-glucosidases. The relative activity toward cardran of 110 K and 210 K ß-glucosidase was 12% and 32%, respectively, of the activity against laminarin. These results suggest that the primary role of 110 K and 210 K ß-glucosidase is the decomposition of cello-oligosaccharides and laminarin to glucose.

**Table 2:** Substrate specificities of 110 K and 210 K ß-glucosidase.

	110 K	210 K
4-MU-ß-Glucoside		
Activity (µmol/min/mg)	15.5	69.9
Km (µM)	24.3±0.5	105±11
Vmax (µmol/min/mg)	22.2±1.5	109±14
Vmax/Km	913	1038
Ki for Gluconolactone (µM)	3.66±0.47	8.10±0.20
Type of inhibition	competitive	mixed
4-MU-ß-Galactoside		
Activity (µmol/min/mg)	5.36	23.6
Km (µM)	753±23	199±5.0
Vmax (µmol/min/mg)	7.33±0.5	29.2±1.6
Vmax/Km	9.73	147
Ki for Gluconolactone (µM)	3.37±0.33	0.884±0.19
Type of inhibition	competitive	competitive
Cellobiose		
Activity (µmol/min/mg)	7.17	110
Km (µM)	0.88±0.17	1.93±0.15
Vmax (µmol/min/mg)	9.07±1.8	156±14
Vmax/Km	10.3	81
Ki for Gluconolactone (µM)	8.08±1.05	17.0±0.30
Type of inhibition	noncompetitive	mixed
Lichenan		
Activity (µmol/min/mg)	1.81	18.9
Km (µM)	1.55±0.10	1.17±0.05
Vmax (µmol/min/mg)	4.93±0.3	40.6±1.1
Vmax/Km	3.18	34.7
Ki for Gluconolactone (µM)	187±20	3.66±0.09
Type of inhibition	noncompetitive	noncompetitive
Laminarin		
Activity (µmol/min/mg)	2.18	36.8
Km (µM)	0.252±0.03	0.264±0.01
Vmax (µmol/min/mg)	2.67±0.3	55.7±5.0
Vmax/Km	10.6	211
Ki for Gluconolactone (µM)	81.1±2.9	10.3±0.80
Type of inhibition	noncompetitive	noncompetitive

### Mode of Inhibition of ß-glucosidase by D-glucono-1,5-lactone

D(+)-Glucono-1,5-lactone (gluconolactone) exhibited mixed-type inhibition of the hydrolysis of *p*-nitrophenyl ß-glucoside by almond ß-glucosidase [34], whereas it is a reversible competitive inhibitor of ß-glucosidases from insects [35] and fungi [36]. In contrast, ß-glucosidase from the Formosan subterranean termite was not inhibited by 10 mM gluconolactone, whereas the activity was strongly inhibited by conduritol B epoxide (IC_50_∶0.6 mM) [36]. Inhibition modes of gluconolactone of the hydrolysis of synthetic and natural substrates by 110 K and 210 K ß-glucosidase were compared (Table 2, Figure S6, S7). Gluconolactone showed different modes of inhibition of the hydrolysis of these substrates. In particular, gluconolactone showed mixed inhibition of the hydrolysis of 4MU-ß-glucoside and cellobiose by 210 K ß-glucosidase and was similar to almond ß-glucosidase, whereas hydrolysis of 4MU-ß-galactoside was inhibited in a competitive manner and that of lichenan and laminarin was inhibited in a noncompetitive manner. In contrast, hydrolysis of 4MU-ß-glucoside and 4MU-ß-galactoside by 110 K ß-glucosidase was inhibited in a competitive manner, whereas the hydrolysis of the natural substrates, cellobiose, lichenan, and laminarin was inhibited in a noncompetitive manner. Compared with the hydrolysis of synthetic substrates and cellobiose, a much higher concentration of gluconolactone was required for inhibition of the hydrolysis of lichenan and laminarin by 110 K ß-glucosidase. Unlike ß-glucosidases from the Formosan subterranean termite [37] and *Streptomyces* spp. [38], conduritol B epoxide exhibited very weak inhibitory activities against 110 K and 210 K ß-glucosidase. Conduritol B-sulfate (10 mM) inhibited 48% and 46%, respectively, of the activity toward 4MU-ß-glucoside and laminarin by 110 K ß-glucosidase. The hydrolysis of 4MU-ß-glucoside and laminarin by 210 K ß-glucosidase was inhibited 20% and 42%, respectively, by 10 mM conduritol B sulfate. The final product, glucose (up to 0.2 M), did not inhibit hydrolysis of 4MU-ß-glucoside by either of the ß-glucosidases.

### Synergistic Effect of Cellulases on Glucose Production from Filter Paper

**Figure 4:**
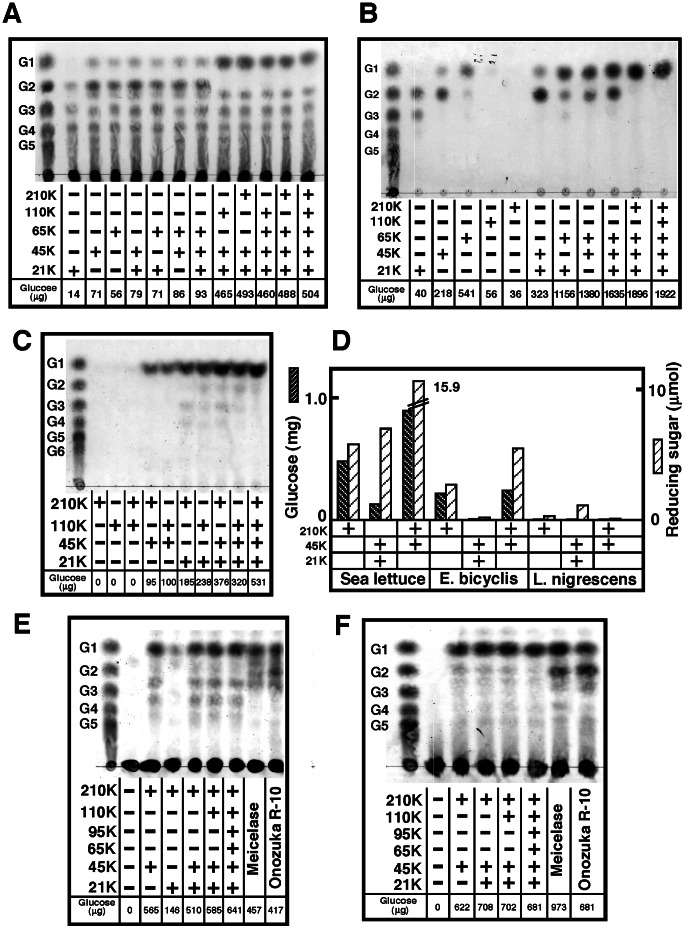
Hydrolysis of CMC, filter paper, and seaweeds by the synergistic action of cellulases and ß-glucosidases. (**A**) CMC (1 mL, 1% in 50 mM acetate, pH 5.5) was incubated with various combinations of purified enzymes (2 µg) as indicated at 37°C for 1 h. Reaction products were analyzed by TLC. (**B**) Filter paper (60 mg) was digested with various combinations of purified enzymes (10 µg) as indicated at 37°C for 48 h, and reaction products were analyzed by TLC. (**C**) Filter paper (60 mg) was digested with 21 K and 45 K cellulase (2 µg) in the presence of 110 K or 210 K ß-glucosidase (2 µg) at 37°C for 16 h. Reaction products were analyzed by TLC. (**D**) Seaweed, sea lettuce (*Ulva pertusa*), *Eisenia bicyclis,* and *Lessonia nigrescens* (20 mg in 50 mM acetate, pH 5.5) were incubated with purified enzymes (10 µg) at 37°C for 24 h. Glucose and reducing sugar content were then determined. (**E, F**) TLC analysis of reaction products of sea lettuce treated with purified enzymes or *Trichoderma* cellulase. Control sea lettuce (**E**) and sea lettuce treated with steam explosion (**F**) (20 mg in 50 mM acetate, pH 5.5) were incubated with purified cellulase (20 µg) in the presence of ß-glucosidase (20 µg) at 37°C for 15 h. As controls, 50 µg of *Trichoderma* cellulases, meicelase, and onozuka R-10 were used. The data shown are from one of three independent experiments with similar results.

Sea hare cellulases can produce glucose from CMC and filter paper without cellobiohydrolase or ß-glucosidase, unlike the *Trichoderma* cellulolytic system [7]. Glucose release from CMC increased markedly following addition of ß-glucosidase. A synergistic effect of 45 K and 65 K cellulase on the production of glucose from filter paper was observed, although no effect was observed on CMC digestion with these enzymes (Figure 4A, 4B). When filter paper (60 mg) was digested with 10 µg of 45 K or 65 K cellulase at 37°C for 48 h in separate tubes, the total glucose released from the filter paper in each reaction was 759 µg (218+541 µg). However, when filter paper was digested with 45 K and 65 K cellulase in the same tube, there was an approximate 2-fold increase in glucose production (1380 µg). Similarly, when filter paper was digested with 21 K, 45 K, and 65 K cellulase in the same tube, there was a 2-fold increase in glucose production compared to reactions in separate tubes. The addition of 210 K or 110 K ß-glucosidase resulted in complete hydrolysis of cellobiose to glucose. To identify the best combination of ß-glucosidases and cellulases to maximize glucose productivity, the glucose-producing activities of the various enzyme mixtures on filter paper were compared (Figure 4C). Filter paper (60 mg) was hydrolyzed to glucose by a pair consisting of either 21 K or 45 K cellulase together with either 210 K or 110 K ß-glucosidase (2 µg of enzyme) at 37°C for 24 h. Compared with the same reaction using all the enzymes (0.531 mg of glucose production), approximately 70% of the glucose productivity was obtained when 3 enzymes, 210 K ß-glucosidase (1.4 units), 21 K (0.036 units), and 45 K cellulase (0.34 units), were used.

### Saccharification of Seaweeds

First, to determine the type of seaweed that can be easily degraded by sea hare enzymes, 5 types of seaweed, dried powder (20 mg) of *Undaria pinnatifida*, *Saccharina angustata*, *Eisenia bicyclis*, *Sargassum fusiforme*, and *Lessonia nigrescens*, were incubated in 1 mL of 50 mM acetate buffer (pH 5.5) containing 210 K ß-glucosidase, 21 K +45 K cellulase, or 210 K +45 K +21 K enzymes at 37°C for 28 h. When the 210 K, 45 K, and 21 K enzymes were incubated together, the seaweeds digested to glucose by this treatment were sea lettuce and *Eisenia bicyclis* (Figure 4D). Glucose was not produced from *Undaria pinnatifida*, *Saccharina angustata*, *Sargassum fusiforme*, or *Lessonia nigrescens* by this treatment. Sea lettuce was the best substrate for sea hare cellulolytic enzymes among the seaweeds examined. Subsequently, sea lettuce (*U. pertusa*) was incubated with various combinations of sea hare digestive enzymes or commercially available cellulases from *Trichoderma* sp. (Figure 4E). Dried sea lettuce powder (20 mg dry weight) was incubated with 1 mL of 50 mM acetate buffer (pH 5.5) containing sea hare enzymes (20 µg of each enzyme) at 37°C for 15 h. When all the sea hare enzymes were used in combination, 0.641±0.021 mg of glucose was released from the sea lettuce. However, almost the same amount (0.565±0.01 mg) of glucose was released by hydrolysis with the pair comprising 45 K cellulase and 210 K ß-glucosidase. When the *Trichoderma*-derived cellulases (0.1 mg) meicelase or onozuka R-10 were incubated with sea lettuce, 0.457 and 0.417 mg of glucose was produced, respectively.

Steam explosion is known to be an effective pretreatment of lignocellulosic materials to increase their digestivity by cellulases [5]. The effect of steam explosion on enzymatic digestion of sea lettuce was also examined (Figure 4F). Although glucose production by *Trichoderma* enzymes was enhanced (1.5–2.0 fold) following pretreatment by steam explosion, digestion by sea hare enzymes was not significantly affected by steam explosion.

## Discussion

Lignocellulose-degrading systems in microorganisms such as *Clostridium thermocellum* [20] and *T. reesei* [6], [7] are well characterized. However, the entire saccharification system of marine invertebrates feeding on macro- and micro-algae has not been fully clarified. Although genome and transcriptome analyses are useful methods to gain an overall understanding of the glucanase genes required for cellulose digestion [21], [39], their enzymatic characteristics such as precise cleavage specificity and synergistic effects cannot be accurately determined from sequence data. Comprehensive analyses of glucanases in digestive juice at the enzyme level are necessary to understand the entire polysaccharide digestion system.

In an effort to clarify the cellulose digestion system in seaweed and to screen for useful glucanases, we purified proteins possessing CMC or 4MU-ß-glucoside hydrolyzing activities from the digestive fluid of the sea hare (*A. kurodai*). We purified 4 cellulases (21 K, 45 K, 65 K, and 95 K) and 2 ß-glucosidases (110 K and 210 K) to a homogeneous state. The synergistic effects of various glucanases on cellulose digestion were then investigated using purified enzymes. All 4 cellulases hydrolyzed CMC, filter paper, and lichenan. The effect on the viscosity of CMC and the cleavage specificity toward natural substrates indicated that the 21 K, 45 K, 65 K, and 95 K cellulase are endo-ß-1,4-glucanase. However, there are some differences in cleavage specificity toward cello-oligosaccharides. These results suggested that 21 K, 45 K, and 65 K cellulase recognize cellulose by the 6, 4, and 3 units of glucose, respectively. Although 95 K cellulase possesses ß-glucosidase activity toward oligosaccharides, the possibility of contamination with ß-glucosidase cannot be excluded. Further analysis of the cleavage specificity of 95 K cellulase is necessary. When these enzymes were incubated with filter paper, cellobiose, cellotriose, and cellotetraose were produced by 21 K cellulose; cellobiose, and glucose were produced by 45 K and 65 K cellulose; and glucose was produced by 95 K cellulase. Endo-ß-1,4-glucanases derived from crustaceans and insects are also active in hydrolyzing cellulose to glucose [14], [40]–[43]. These enzymes seem to possess characteristic properties of cellobiohydrolase and ß-glucosidase.

The molecular mass of numerous termite ß-glucosidases is 50–60 kDa [39], [44], [45]. Eukaryotic ß-glucosidases with a molecular mass larger than 60 kDa have been identified at the protein level in the herbivorous gecarcinid land crab *Gecarcoidea natalis* (130 kDa) [40], the filamentous fungus *Acremonium persicinum* (128 kDa) [46], *T. reesei* (114 kDa and 71 kDa) [47], (81.6 kDa) [48], and the fungus-growing termite *Macrotermes barneyi* (110 kDa, MbmgBG2) [49]. 210 K ß-glucosidase is the largest enzyme among reported eukaryote β-glucosidases. The 2 sea hare ß-glucosidases preferentially hydrolyze the ß-1,4 and ß-1,3 bonds of glucose. Reaction with endoglucanase and ß-glucosidase is necessary for efficient production of glucose from CMC. These results suggest that both ß-glucosidases preferentially hydrolyze the short cellulose fragment produced by endoglucanase digestion. In contrast, both β-glucosidases hydrolyze laminarin and lichenan to glucose in the absence of endoglucanases, although the activity of 110 K ß-glucosidase toward both substrates is lower than that of 210 K ß-glucosidase. Furthermore, both ß-glucosidases also exhibit ß-galactosidase and lactase activities.

Studies on the inhibition of ß-glucosidases by gluconolactone clearly indicated differences in the catalytic properties of 110 K and 210 K ß-glucosidase. There are differences in inhibitor sensitivity and inhibition manner between 110 K and 210 K ß-glucosidase. In particular, 210 K ß-glucosidase is more sensitive against gluconolactone in reaction with lichenan (Ki: 3.66 µM) and laminarin (Ki: 10.3 µM) than 110 K ß-glucosidase (Ki: 187 µM, 81 µM). Gluconolactone showed mixed inhibition of the hydrolysis of 4MU-ß-glucoside and cellobiose by 210 K ß-glucosidase. In contrast, hydrolysis of laminarin and lichenan by the 210 K enzyme was inhibited in a noncompetitive manner. Hydrolysis of 4MU-ß-galactoside by the 210 K enzyme was inhibited in a competitive manner. Moreover, hydrolysis of 4MU-ß-glucoside and 4MU-ß-galactoside by 110 K ß-glucosidase was inhibited in a competitive manner. As with 210 K ß-glucosidase, hydrolysis of both laminarin and lichenan was inhibited in a noncompetitive manner. However, inhibition of the hydrolysis of cellobiose showed noncompetitive inhibition. These results suggest that (i) 110 K and 210 K ß-glucosidase possess 2 catalytic sites, although identification of 2 active sites is necessary, and (ii) the structure of the active site and subsite of 210 K and 110 K ß-glucosidase is similar but not identical.

The most striking finding in the present investigation is the similarity of sea hare 210 K ß-glucosidase with human LPH in terms of molecular mass, amino acid sequences, and catalytic properties. Human LPH is a small intestinal disaccharidase essential for hydrolysis of lactose in milk [31]. LPH is a ß-glucosidase with broad substrate selectivity that can hydrolyze ß-glucosides and ß-galactosides through 2 separate active sites, the lactase site and the phlorizin hydrolase site [50]. Cellobiose is also a substrate for LPH [51]. Recently, ß-glucosidase cDNA encoding a 107 kDa protein belonging to GHF 1 was cloned from the common Japanese brackish water clam *Corbicula japonica* [23], although its enzymatic properties have not been clarified. The predicted amino acid sequence of brackish water clam ß-glucosidase also showed high similarity with LPH. Two catalytically important glutamic acid residues and amino acid residues involved in substrate binding and conserved in GHF-1 were completely conserved in the GHF1 domain of brackish water clam ß-glucosidase and LPH. On the other hand, substrate specificity and the method of inhibition against gluconolactone suggested that sea hare 210 K ß-glucosidase possesses 2 active sites like LPH. Further analysis is necessary to understand the functional share of 110 K and 210 K ß-glucosidase.

It is currently believed that 3 types of glycoside hydrolases, comprising endoglucanases, cellobiohydrolases, and ß-glucosidases, are required for efficient decomposition of cellulose. However, 1 pair of sea hare enzymes, 45****K endoglucanase and 210****K ß-glucosidase, exhibited a significant glucose release capability from sea lettuce. This capability was comparable with that of commercial fungal cellulases. It is highly likely that the high glucose-releasing activity of the 2-enzyme mixture is because of the unique properties of both enzymes, as mentioned above. 45****K cellulase, the most abundant cellulase in digestive fluid of the sea hare, exhibits cellobiohydrolase and lichenase activities, in addition to ß-1,4-endoglucanase activity. 210****K ß-glucosidase hydrolyzes not only the ß-1,4 bond of glucose but also the ß-1,3 bond within laminarin. 210****K ß-glucosidase possesses broad cleavage specificity. Unlike cellulose, laminarin and lichenan are digested to glucose by 210****K ß-glucosidase in the absence of endoglucanases. Our results indicate that laminarin is an important source of glucose, as well as cellulose, in sea lettuce.

All cellulases and ß-glucosidases purified from sea hare digestive fluid are glycoproteins, suggesting that these enzymes are produced by the sea hare itself and not by gut-resident bacteria. However, it is important to note that gut-resident protists can also synthesize glycoproteins. Further analyses of the origin of these enzymes are therefore necessary.

Taken together, our findings provide important new evidence showing that the sea hare can produce glucose from sea lettuce using a simpler enzyme system than the fungal and microbial lignocellulose digestion system. With respect to biofuel production, the present study constitutes an important step in our effort to identify essential enzymes that can release fermentable simple sugars from seaweeds. The sea hare digestion system may thus provide useful clues for the establishment of an artificial process for seaweed biomass saccharification.

## Materials and Methods

### Materials

The sea hare (*A. kurodai,* body length, 20–25 cm) and sea lettuce (*U. pertusa*) were collected on the coast of Naruto, Japan, during April–July. Sea hare and sea lettuce are not protected in this area. No specific permissions were required since collection of these species is allowed. Each sea hare was cooled in an ice bath, and digestive fluid was obtained from the gastric lumen by squeezing the stomach after dissection. The digestive fluid was filtered through 2 layers of gauze to remove undigested seaweed. Approximately 500 mL of digestive fluid was collected from 10 animals. The digestive fluid was then fractionated with ammonium sulfate (0–60% saturation). After centrifugation at 20,000×*g* for 15 min, the precipitate was dissolved in 20 mM Tris-HCl buffer (pH 7.0) and dialyzed against the same buffer at 4°C. The dialyzate was centrifuged at 20,000×*g* for 10 min. The resulting supernatant was concentrated by ultrafiltration (polyethersulfone membrane, Millipore, Billerica, MA) and stored at −30°C until use. The sea lettuce was washed with water, dried at 50°C, and then minced using a Waring blender. Dried seaweeds (*U. pinnatifida*, *S. angustata*, *E. bicyclis*, *S. fusiforme*, and *L. nigrescens*) were purchased from a local grocery store and minced using a Waring blender.

Carboxymethylcellulose (CMC, sodium salt, low viscosity), laminarin (ß-1,3∶1,6-glucan) from *Laminaria digitata*, 4-methylumbelliferyl (4MU)-ß-D-glucoside, 4MU-ß-D-galactoside, and 4MU-ß-D-xyloside were purchased from Sigma-Aldrich (St, Louis, MO). Lichenan was obtained from Megazyme (Bray, Ireland). Cellotriose, cellotetraose, and cellohexaose were obtained from Carbosynth (Compton, UK). D(+)-cellobiose, D(+)-glucono-1,5-lactone, and Glucose CII Test Wako were obtained from Wako Pure Chemicals (Osaka, Japan). DEAE-Sepharose™ (fast flow), CM-Sepharose™ (fast flow), phenyl-Sepharose (HiLoad™ 16/10), Sephacryl S-100, Sephacryl S-200, and Mono-Q HR5/5 were obtained from GE Healthcare (Uppsala, Sweden). Hydroxyapatite was purchased from Seikagaku Kogyo (Tokyo, Japan). A peroxidase-labeled lectin kit was purchased from J-OIL MILLS (Tokyo, Japan). All other chemicals used were of analytical grade.

### Enzyme Assay

Cellulase, lichenase, and laminarinase activities were assayed using CMC, lichenan, and laminarin as substrates, respectively, as described previously [14]. The quantities of reducing sugars liberated by the hydrolysis of substrates were determined by the method of Nelson and Somogyi [52]. One unit of enzyme activity was defined as that amount of enzyme that liberates reducing sugars equivalent to 1 µmol of glucose per min at 37°C. ß-glucosidase, ß-galactosidase, ß-mannosidase, and ß-xylanase activities were assayed using the 4MU substrates 4MU-ß-glucoside, 4MU-ß-galactoside, 4MU-ß-mannoside, and 4MU-ß-xyloside, respectively, as described previously [14]. Released 4-methylumbelliferone was measured fluorometrically (excitation 365 nm, emission 450 nm). One unit (U) was defined as the activity that produced 1 µmol of 4-methylumbelliferone per min at 37°C. Glucose liberated by hydrolysis of CMC, lichenan, laminarin, filter paper, and sea lettuce was determined by the Glucose CII Test Wako kit using glucose oxidase. Protein concentration was determined by the Bradford method using BSA as the standard [53]. Km, Vmax, and Ki were determined using a Lineweaver–Burk plot. When gluconolactone showed mixed type of inhibition, Ki was determined by the method of Cornish–Bowden [54]. The kinetic data and SDs were czlculated from at least three separate experiments.

### Purification of Cellulases from Digestive Fluid

All purification procedures were performed at 4°C, and cellulase (endo-ß-1,4-glucanase) activity was measured using carboxymethylcellulose unless otherwise stated. A frozen ammonium sulfate fraction from 300 mL of digestive fluid, prepared as mentioned above, was thawed and centrifuged at 12,000×*g* for 10 min. The supernatant was applied to a CM-Sepharose column (2.5×20 cm) equilibrated with 20 mM Tris-HCl (pH 7.0) and washed with the same buffer. The fraction that passed through the column contained 20–30% of the total cellulase activity of the ammonium sulfate fraction. It was concentrated by ultrafiltration and stored at −30°C until use. Proteins bound to the CM-Sepharose column were eluted using a linear gradient of NaCl (0–0.3 M) in the same buffer. The fractions possessing cellulase activity were concentrated by ultrafiltration, and ammonium sulfate was then added to the concentrate to produce the final concentration of 1 M. Following centrifugation, the supernatant was applied to a phenyl-Sepharose (HiLoad^™^ 16/10) column equilibrated with 20 mM Tris-HCl (pH 7.0) containing 1 M ammonium sulfate and washed with the same buffer. Bound proteins were eluted using a linear gradient of ammonium sulfate (1–0 M). The elution profile of proteins was confirmed by SDS-PAGE [26]. Eight fractions (A–H) were pooled, concentrated, and applied to a Sephacryl S-100 column (2.0×105 cm) equilibrated with 20 mM Tris-HCl (pH 7.0) containing 0.1 M NaCl. The A fraction was eluted as a single peak (A fraction) possessing cellulase activity. Cellulase activity was not detected in the B, C, or D fractions. The E fraction was separated into 3 peaks (E-I, E-II, E-III) using Sephacryl S-100 gel filtration. The E-I, E-II, and E-III fractions contained 65 kDa, 35 kDa, and 32 kDa of protein, respectively. Cellulase activity was detected in the E-I fraction but not in the E-II or E-III fraction. The F fraction was further purified by Sephacryl S-100 gel filtration, but a sufficient amount of protein possessing cellulase activity was not obtained. The G fraction was separated into 2 peaks (G-I and G-II) by Sephacryl S-100 gel filtration. The G-I fraction possessed cellulase activity and contained 45 kDa of protein as a major component. The G-II fraction did not exhibit cellulase activity and contained 52 kDa of protein. The H fraction was eluted as a single peak comprising 45 kDa of protein following Sephacryl S-100 gel filtration and possessed cellulase activity.

A fraction obtained from a CM-Sepharose column was applied to a DEAE-Sepharose (2.5×20 cm) column equilibrated with 20 mM Tris-HCl buffer (pH 7.0), and bound proteins were eluted using a linear gradient of NaCl (0–0.5 M). ß-glucosidase activity was eluted as 2 peaks (DE-I and DE-II fractions). The DE-I fraction was applied to a phenyl-Sepharose column and eluted using a linear gradient of ammonium sulfate (1–0 M). The fractions possessing ß-glucosidase activity were concentrated by ultrafiltration and subjected to Sephacryl S-200 (3.0×95 cm) gel filtration. The first peak possessed ß-glucosidase activity, and a protein of 110 kDa was detected by SDS-PAGE. The 110 kDa ß-glucosidase fraction was then applied to a hydroxyapatite column (1.0×4.0 cm) equilibrated with 20 mM acetate (pH 6.5) and eluted using a linear gradient of sodium phosphate (0–0.2 M). The fractions possessing ß-glucosidase activity and containing a single 110 kDa protein were pooled and concentrated. The DE-II fraction was applied to a phenyl-Sepharose column, and bound proteins were eluted using a linear gradient of ammonium sulfate (1–0 M). The fractions with ß-glucosidase activity were concentrated and subjected to Sephacryl S-200 gel filtration. The first peak (“a” fraction) and second peak (“b” fraction) possessed ß-glucosidase and cellulase activity, respectively. Proteins of 210 kDa and 95 kDa were detected in the “a” and “b” fraction, respectively. The 210 kDa ß-glucosidase fraction was further purified by Mono-Q column chromatography. The “a” fraction was dialyzed against a 20 mM acetate buffer (pH 6.5) and subjected to a Mono-Q column and eluted using a linear gradient of NaCl (0–0.5 M). The fractions possessing ß-glucosidase activity were pooled and concentrated.

### Analysis of Degradation Products by Thin Layer Chromatography (TLC)

TLC for analysis of the degradation products of CMC, filter paper, lichenan, laminarin, disaccharide, and cello-oligosaccharides was performed using TLC Silica gel 60F plates (Merck KGaA, Darmstadt, Germany), and the products were detected using orcinol-sulfuric acid as described previously [55].

### Measurement of Viscosity

The effect of enzymatic activity on the viscosity of CMC was determined by comparing the flow rate in an Ostwald viscometer with that of a control CMC sample as described previously [14].

### Sequence Analysis

Purified enzymes separated by SDS-PAGE were electroblotted onto PVDF membranes (Immobilon™, 0.45 mm, Millipore, Bedford, MA) according to the manufacturer’s instructions. The protein band stained with Ponceau 3R was applied to an automated protein sequencer (Shimadzu PPSQ-10, Kyoto, Japan), and the amino-terminal sequence was determined. To determine the internal sequence, the protein band was digested with lysyl endopeptidase [56] and the released peptides were purified by reversed-phase high-performance liquid chromatography as described previously [57]. Amino acid sequences were analyzed using an automated protein sequencer (Shimadzu PPSQ-10, Kyoto, Japan).

### Analysis of Glycoprotein by Lectin Blot

Lectin blot of glycoprotein was carried out using horseradish peroxidase-labeled lectin (ConA, LCA, PHA-E4, PNA, RCA120, and WGA) according to the manufacturer’s protocol. Purified cellulases and ß-glucosidases were blotted onto a PVDF membrane after SDS-PAGE and the membrane was incubated with 20 µg/mL of lectin for 1 h at room temperature. A lectin-reactive band was then detected using 3,3′-diaminobenzidine tetrahydrochloride.

### Steam Explosion Pretreatment of Sea Lettuce

Steam explosion pretreatment was performed in a batch apparatus equipped with a 2 L high pressure reactor (Steam explosion apparatus NK-2L; Japan Chemical Engineering and Machinery Co Ltd., Osaka, Japan). Fresh sea lettuce was introduced into the reactor and exposed to saturated steam at a pressure of 20 atm (214°C) for a steaming time of 5 min, as described previously [58].

## Supporting Information

Figure S1 **Flow sheet of the purification of cellulases (Cel) and ß-glucosidases (BGL) from digestive fluid of the sea hare** ***Aplysia kurodai.***
(TIFF)

Figure S2 **Purification of 21 K, 45 K, and 65 K cellulase from digestive fluid of the sea hare.** Cellulases in digestive fluid of the sea hare were purified by ammonium sulfate fractionation (0–60%), CM-Sepharose, phenyl-Sepharose, and Sephacryl S-200 or S-100 column chromatography, as described in Materials and Methods. The fractions (A, E, G, and H) possessing cellulase activity and separated using phenyl-Sepharose chromatography were further purified by gel filtration. The E fraction was fractionated into E-I, E-II, and E-III by Sephacryl S-200 gel filtration. The G fraction was separated into G-I and G-II by Sephacryl S-200 gel filtration. The H fraction was purified by Sephacryl S-200 gel filtration. The fraction indicated by the horizontal bar was concentrated and analyzed by SDS-PAGE. Fractions A and E–I contained 21 kDa and 65 kDa of cellulase (21****K and 65****K cellulase), respectively, as a single protein. G–I and H fractions contained 45 kDa of cellulase (45****K cellulase).
(TIFF)

Figure S3 **Purification of 210 K and 110 K ß-glucosidase and 95 K cellulase using DEAE-Sepharose and Sephacryl S-200.** 210****K and 110****K ß-glucosidase and 95****K cellulase in the CM-Sepharose unbound fraction were purified by DEAE-Sepharose chromatography **(A)** and fractionated into DE-I and DE-II fractions. The DE-I and DE-II fractions were subjected to phenyl-Sepharose followed by Sephacryl S-200 gel filtration **(B** and **C).** The DE-I fraction was separated into 3 peaks following gel filtration. The first peak possessed ß-glucosidase activity. The DE-II fraction was separated into fractions possessing ß-glucosidase activity (“a” fraction) and cellulase activity (“b” fraction).
(TIFF)

Figure S4 **Purification of 110 K and 210 K ß-glucosidase using hydroxyapatite and Mono-Q chromatography. (A)** 110****K ß-glucosidase (Figure S3B) was further purified by hydroxyapatite chromatography. Fraction #42–70 contained 110 kDa of ß-glucosidase as shown by SDS-PAGE (inset). **(B)** The “a” fraction (Figure S3C) was further purified by Mono-Q chromatography. Fraction #25–30 contained 210 kDa of ß-glucosidase as shown by SDS-PAGE (inset). The fractions indicated by the horizontal bar were concentrated and dialyzed against a 20 mM Tris-HCl buffer (pH 7.0).
(TIFF)

Figure S5 **Lectin blot of purified cellulases and ß-glucosidases.** The purified enzymes were boiled in a 2% SDS solution containing 10% ß-mercaptoethanol and then resolved by electrophoresis through a 12.5% gel. Protein was detected using Coomassie Brilliant Blue (CBB) and horseradish peroxidase-labeled lectin (WGA, ConA, PNA and LCA), as described in Materials and Methods.
(TIFF)

Figure S6 **Inhibition of ß-glucosidase by D-glucono-1,5-lactone (GL) using 4MU-ß-glucoside, 4MU-ß-galactoside, and cellobiose as substrates.** 210****K (A, C, E) and 110****K (B, D, F) ß-glucosidase against 4MU-ß-glucoside (A, B), 4MU-ß-galactoside (C, D), and cellobiose (E, F).
(TIFF)

Figure S7 **Inhibition of ß-glucosidase by D-glucono-1,5-lactone (GL) using lichenan and laminarin as substrates.** 210****K (A, C) and 110****K (B, D) ß-glucosidase against lichenan (A, B) and laminarin (C, D).
(TIFF)
