# Supplementary figures and images for: Comprehensive Enzymatic Analysis of the Cellulolytic System in Digestive Fluid of the Sea Hare Aplysia kurodai. Efficient Glucose Release from Sea Lettuce by Synergistic Action of 45 kDa Endoglucanase and 210 kDa ß-Glucosidase
Source: PLoS One. 2013 Jun 6;8(6):e65418. doi: 10.1371/journal.pone.0065418 (PMC3675134; doi:10.1371/journal.pone.0065418)

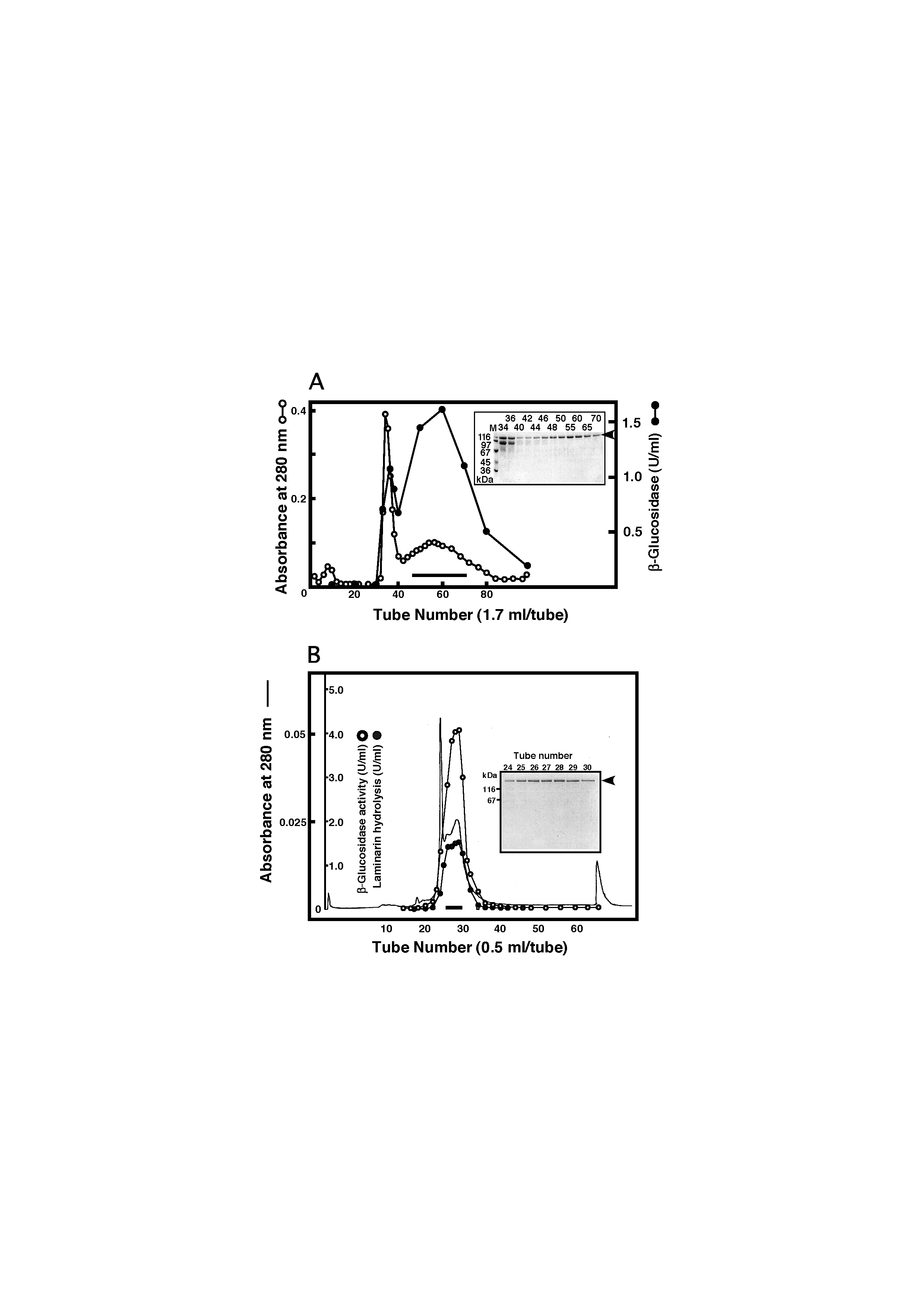

Supplement: Figure S4 — Purification of 110 K and 210 K ß-glucosidase using hydroxyapatite and Mono-Q chromatography. (A) 110K ß-glucosidase (Figure S3B) was further purified by hydroxyapatite chromatography. Fraction #42–70 contained 110 kDa of ß-glucosidase as shown by SDS-PAGE (inset). (B) The “a” fraction (Figure S3C) was further purified by Mono-Q chromatography. Fraction #25–30 contained 210 kDa of ß-glucosidase as shown by SDS-PAGE (inset). The fractions indicated by the horizontal bar were concentrated and dialyzed against a 20 mM Tris-HCl buffer (pH 7.0). (TIFF) [file pone.0065418.s004.tiff]

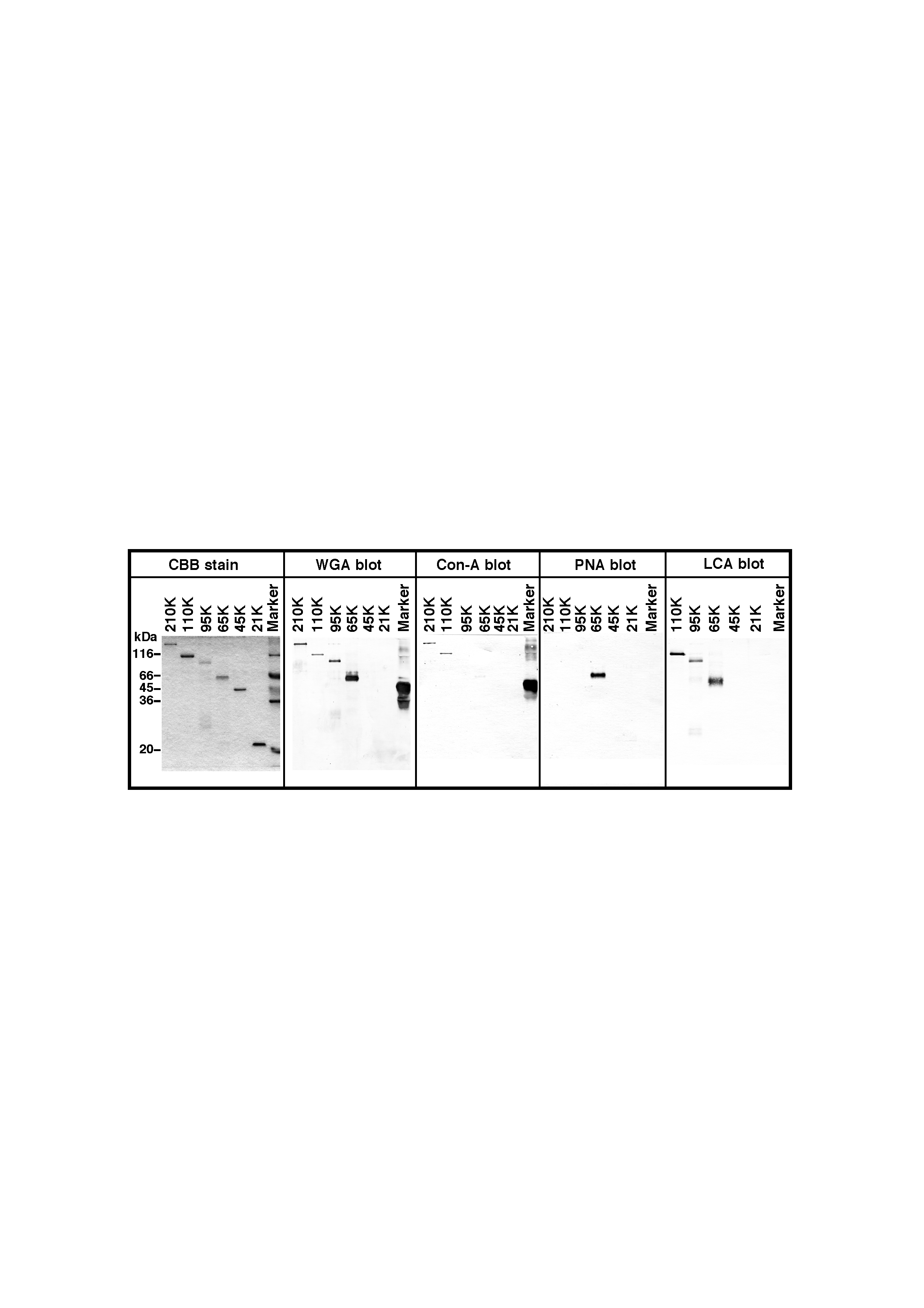

Supplement: Figure S5 — Lectin blot of purified cellulases and ß-glucosidases. The purified enzymes were boiled in a 2% SDS solution containing 10% ß-mercaptoethanol and then resolved by electrophoresis through a 12.5% gel. Protein was detected using Coomassie Brilliant Blue (CBB) and horseradish peroxidase-labeled lectin (WGA, ConA, PNA and LCA), as described in Materials and Methods. (TIFF) [file pone.0065418.s005.tiff]
